# Nicotinic Acid Long-Term Effectiveness in a Patient with Bipolar Type II Disorder: A Case of Vitamin Dependency

**DOI:** 10.3390/nu10020134

**Published:** 2018-01-27

**Authors:** Bo H. Jonsson

**Affiliations:** Centre for Psychiatry Research, Department of Clinical Neuroscience, Karolinska Institutet, & Stockholm Health Care Services, Stockholm County Council, Norra Stationsgatan 69, SE-113 64 Stockholm, Sweden; bo.jonsson@ki.se; Tel.: +46-708-200-034

**Keywords:** nicotinic acid, niacin, vitamin B_3_, bipolar disorder, anxiety, mood

## Abstract

Nicotinic acid (NA), often called niacin, a form of vitamin B_3_, is a water-soluble nutrient found in animal and vegetarian foods. Vitamin B_3_ for healthy people is considered to be needed in doses of less than 20 mg daily. In higher doses, NA has been described to be beneficial in some patients with psychiatric disorders. This report describes a male patient with bipolar type II disorder who for many years had been treated with lithium and other medications applied in affective disorders. These pharmacological drugs had beneficial effects but were at times insufficient. When the patient was prescribed NA, he experienced a comparatively strong effect. Slowly it was discovered that the patient could lower and cease all medications except NA. For over 11 years he has been stable and calm with NA and currently takes 1 g three times daily. When not taking NA, he consistently became anxious and depressed within 2–3 days. The resumption of NA resulted in a normal state usually within 1 day. This finding has been described as a vitamin dependency. The paper discusses possible mechanisms for the effect of NA in this patient. Further studies are needed to investigate the prevalence of vitamin B_3_ dependency and the biochemical explanations for this phenomenon.

## 1. Introduction

Bipolar disorder has a lifetime prevalence rate of around 2% [1]. If the patient has had at least one manic episode, this is called bipolar disorder type I. If the patient has had hypomania but not mania, we speak of bipolar type II. 

Most patients with a bipolar disorder are treated with pharmacological drugs, usually lithium, antiepileptics or antipsychotics. These medications are often effective alone or combined. However, side effects, complicating diseases, non-response and the opinion of the patient are to be respected. 

NA is a form of vitamin B_3_. It is required in daily doses lower than 20 mg to avoid the deficiency disease pellagra. That pellagra often resulted in different psychiatric states, sometimes of manic-depressive type, was described [2] before it was established to be a nutritional deficiency syndrome [3,4]. Thereafter it was demonstrated that NA helped patients with mental symptoms of pellagra [5]. Positive reports were later described for NA treatment in schizophrenic patients [6].

In PubMed and the Web of Science, there seem to be no cases published with a positive result from using NA in bipolar patients. However, there is one case report in which treatment with NA resulted in an acute manic psychotic episode [7]. 

## 2. Case Report

The patient (P) was a man born in 1945. 

Heredity: P’s grandmother had been hospitalised for a psychiatric disorder. His sister suffered from major depressions, had been treated with electroconvulsive therapy and later on committed suicide. P had a son who tried to commit suicide during a depressive episode. His two daughters also had an affective sensitivity with depressions.

Diagnosis: bipolar syndrome, type II; periods of depression and hypomania, episodic.

Patient history: As long as he remembered, P had been depressed most often during March, July and November. During the 1970s, he made four suicidal intoxications with drugs and alcohol. He was in inward treatment for depression twice in Sweden and once in England. During periods in hypomanic states with high efficiency and less sleep, he was never hospitalised. Sometimes he made hasty decisions, and once he married a woman he had only known for 14 days. 

Treatment with lithium in 1974–1976 had a good effect against the depressions. However, he felt he did not have access to his feelings and chose to go off lithium for 6 years. He noted that he had more depressions again. From 1982, he took lithium again with a good effect. Sometimes he tried not to take lithium and relapsed into depressive states. 

As P moved from another city he became my patient in 1991. Except for lithium, he had for years been treated with carbamazepine, antidepressants, anxiolytics and also psychotherapy. In 2005, P was taking lithium, moclobemide, diazepam and zolpidem, all in conventional doses. 

October 2005: P was aggressive, anxious and depressive after problems at his job where he worked as headmaster. He was prescribed Niaspan (nicotinic acid extended release) because the psychiatrist knew NA was sometimes effective in anxiety states [8]. In a few weeks, this was increased to 1000 mg twice a day. November 2005: After 3 weeks, he felt somewhat better and stopped worrying. After 6 weeks, P expressed, “During the last 10 years, since I lost my job last time, I felt like walking in a swamp. Now I feel I walk on solid ground… I was surprised it was such a strong difference. I have no suicidal thoughts, which I usually have had. I am positive, calmer, sleep better and my high blood pressure is normalized”. When stressed at work, he could face his problems adequately. Being positive to Niaspan, he proceeded continuously with 1000 mg morning and evening. 

January 2006: P had stopped diazepam for 4 weeks. He felt better, but not completely well. He was more stable, had no anxiety and less-impulsive aggression and depression, and was able to make plans for the future.

March 2006: P stopped taking zolpidem, and his sleep was good. P started to decrease moclobemide. 

May 2006: P stopped the antidepressant moclobemide. As P was feeling comparatively well, the possibility of reducing lithium was actualized and an agreement to do so slowly was made. 

June 2006: P started reducing lithium.

August 2006: P finished lithium, felt more sensitive (which he thought was positive) and was slightly depressed for 1 month.

October 2006: P described feeling much more alert compared to when he was on the earlier drugs.

February 2008: P reported that he twice forgot to take NA when traveling and once chose not to take it as a test. All three times, after 2–3 days, he became irritated, nervous, restless and itchy. When taking 1000 mg of NA he became better within less than 12 h. 

In June 2011, his wife wondered if he really needed the medication, and P made a test not to take Niaspan. After a week, he felt more irritated, aggressive, anxious, depressive and developed suicidal thoughts. When taking 1000 mg of Niaspan twice daily, he was again in good balance after 2–3 weeks. 

During autumn 2011, Niaspan was taken off the market in Sweden, and the prescription was changed to Tredaptive (1000 mg NA with 20 mg lorapiprant). After increasing to 2000 mg of NA each day, P found this to have a weaker effect than Niaspan; thus the dose was increased to 3000 mg daily. This may also be justified by P’s weight of 105 kg. He then had a good effect with no anxiety, no suicide thoughts and a stable mood. 

January 2013: Tredaptive was taken off the market. Thereafter P was prescribed NA (500 mg) in capsules to be taken two each at breakfast, lunch and dinner. P more often developed a flush, but once used to it, he found no problem with this reaction. The psychiatric effect was continuously positive. 

Side effects from NA: Over 12 years, P experienced only a few and minor adverse effects from NA. Before starting with NA, he had an increased blood pressure, but after some time he found it stabilized. When changing to immediate-release NA in 2013, P had a flush reaction more often. However, he did not find this problematic. Over the years, liver enzymes in blood were normal for this patient. 

The patient has given written consent to this case report.

## 3. Discussion

The course of bipolar disorder varies much over time, both in a single person and between different individuals. This is one reason that long-term case studies may be valuable in studies of bipolar syndromes. One-person trials have different strengths and weaknesses compared to multi-person trials [9]. From a single case, it is not possible to generalize for evaluation and treatment of other patients. However, case reports may aid in developing further hypotheses and in the understanding of mechanisms and therapy. 

In everyday life, we observe that all humans are different in looks and behaviour. However, we also have biochemical individuality [10,11]. This knowledge is crucial in studying effects and side effects of different biochemical substances. 

When NA was prescribed, the purpose was to decrease anxiety. As this effect emerged, it was easy to stop diazepam and zolpidem. However, that the effect in this patient also stabilised his mood was a surprise. Reducing and stopping lithium treatment had not been contemplated when NA was introduced for this patient. For over 11 years, NA has functioned as a mood stabiliser in this patient with bipolar type II disorder. He also described having a generally more positive feeling with NA compared to his previous medications including lithium. Because P ceased taking lithium, he used no other medication.

However, whenever the patient forgot to take NA or decided to test not taking it, he consistently became anxious within 2 to 3 days. When P resumed taking NA, he would usually feel normal within a day. 

The phenomenon that a high-dose vitamin is needed for treatment of a diagnostic entity in some patients has been described as a *vitamin dependency*. This concept is most well known for vitamin B_6_ with respect to a form of epilepsia described in children [12]. The explanation for a higher need of vitamin B_6_ can be related to use of interfering drugs, somatic disease with malabsorption, renal dialysis, an inborn error of metabolism or genetic polymorphism. Vitamin dependency has also been described for vitamins B_1_ [13], B_3_ [14], B_12_ [15], folate [16], D [17] and K [18]. 

What could be the reason for the effect of high-dose NA in this case? Vitamin B_3_ is usually estimated to be necessary in doses of less than 20 mg daily. The statement is correct if the purpose is to prevent the deficiency disease pellagra in the population. However, if some individuals need a much higher dose of this water-soluble molecule, it is reasonable to assume a specific biochemical explanation for this. 

P did not take any other drugs and did not have any known somatic disease that might have affected the metabolism of NA. Additionally, the fact that several people in P’s family had psychiatric disorders suggests that genetic factors may be of etiologic importance in this case. 

High-dose NA is helpful in some patients with different diseases. Most well known is the use of NA in gram doses for hyperlipidaemia [19]. However, NA acts via several pathways. NA is a precursor to nicotinamide (NAM), nicotinamide adenine dinucleotide (NAD), nicotinamide adenine dinucleotide hydrogen (NADH), nicotinamide adenine dinucleotide phosphate (NADP) and nicotinamide adenine dinucleotide phosphate hydrogen (NADPH). These substances participate in the metabolism of cells and their mitochondria. They act as substrates, participate in enzymatic processes, stimulate receptors and interact with genes. NA is converted to NAM, which has benzodiazepine-like actions in the gamma-aminobutyric acid (GABA) system [20,21]. Further, NA affects brain receptors and is involved in the kynurenine pathway, considered to be of interest in bipolar disorder [22]. NA is also neuroprotective [23]. However, if the patient has specific polymorphisms, this may affect NAD biochemistry and the binding and affinity of related molecules. As NAD participates in more than 470 chemical reactions [24], this may have significant importance. Disrupted NAD synthesis may cause NAD deficiency [25]. Lower NAD levels relate to aging, mitochondrial dysfunction, cancer and other age-related diseases [26,27]. Altered expression of sirtuin 1 (SIRT1) has been described in bipolar patients [28,29]. Sirtuins are a form of NAD^+^-dependent histone deacetylases, which regulate different cellular functions. 

The intake of NAD precursors may help to increase levels for optimal NAD biochemical function. This has been studied in animal studies, human cell cultures and in humans [30,31,32]. Several genetic conditions respond to higher doses of NA [33]. How often this may help patients with a bipolar disorder is unknown. Bipolar disorder is a complex psychiatric disease. Both genetic and nongenetic factors are important. The knowledge base is yet not solid for genetic testing, and further work is needed [34].

## 4. Conclusions

The author has been the treating psychiatrist for this bipolar type II patient for 26 years. Twelve years ago, the patient was prescribed NA as an add-on to conventional medication. Successively, the psychopharmacological drugs were terminated. With NA, currently at 1000 mg three times daily, the patient has for over 11 years been stable and in a good mental health condition without any psychiatric drugs. The most probable explanation is that this is related to specific genetic factors. Such a high-dose need of an essential vitamin is sometimes described as a vitamin dependency. Further studies may help us to see if NA dependency occurs in different patient groups. The investigation of possible genetic polymorphisms is needed for the development of knowledge and the individual treatment of patients. 
